# Polyacrylamide-Based Polymers for Slickwater Fracturing Fluids: A Review of Molecular Design, Drag Reduction Mechanisms, and Gelation Methods

**DOI:** 10.3390/gels12020101

**Published:** 2026-01-26

**Authors:** Wenbin Cai, Weichu Yu, Fei Ding, Kang Liu, Wen Xin, Zhiyong Zhao, Chao Xiong

**Affiliations:** 1 College of Chemistry & Environmental Engineering, Yangtze University, Jingzhou 434023, China; 2 CCDC Downhole Operation Company, CNPC, Chengdu 610051, China

**Keywords:** polyacrylamide, synthesis methods, drag reduction, gelation, slickwater fracturing fluid

## Abstract

Slickwater fracturing has become an adopted technology for enhancing hydrocarbon recovery from unconventional, low-permeability reservoirs such as shale and tight formations, owing to its ability to generate complex fracture networks at a low cost. Polyacrylamide and polyacrylamide-based gels serve as key additives in these fluids, primarily functioning as drag reducers and thickeners. However, downhole environments of high-temperature (>120 °C) and high-salinity (>1 × 10^4^ mg/L) reservoirs pose challenges, leading to thermal degradation and chain collapse of conventional polyacrylamide, which results in performance loss. To address these limitations, synthesis methods including aqueous solution polymerization, inverse emulsion polymerization, and aqueous dispersion polymerization have been developed. This review provides an overview of molecular design methods aimed at enhancing performance stability of polyacrylamide-based polymers under extreme conditions. Approaches for improving thermal stability involve synthesis of ultra-high-molecular-weight polyacrylamide, copolymerization with resistant monomers, and incorporation of nanoparticles. Methods for enhancing salt tolerance focus on grafting anionic, cationic, or zwitterionic side chains onto the polymer backbone. The drag reduction mechanisms and gelation methods of these polymers in slickwater fracturing fluids are discussed. Finally, this review outlines research directions for developing next-generation polyacrylamide polymers tailored for extreme reservoir conditions, offering insights for academic research and field applications.

## 1. Introduction

**Figure 1:**
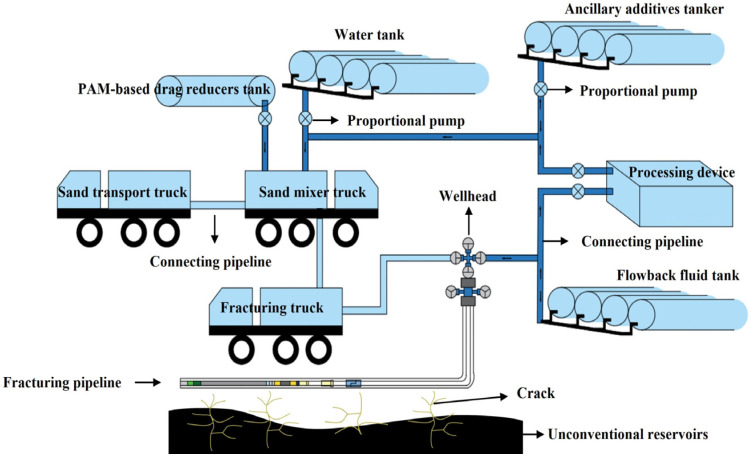
Schematic illustration of slickwater fracturing.

Petroleum and natural gas are vital fossil fuels underpinning modern industrial society and global energy supplies [1,2,3]. These hydrocarbons, however, are predominantly stored in deep geological formations characterized by inherently low porosity and permeability, such as shale and tight sandstone reservoirs [4,5]. Their economic extraction therefore relies heavily on effective stimulation technologies, particularly hydraulic fracturing. Microseismical monitoring data consistently show that, compared to conventional gel-based fracturing fluids, slickwater fracturing generates (Figure 1) more extensive and complex fracture networks within target formations [6,7]. These interconnected networks significantly enhance the effective permeability of the reservoir, creating critical pathways for hydrocarbons to flow to the wellbore and ultimately leading to substantially improved recovery rates.

Since its pioneering large-scale application in the Barnett Shale play, slickwater fracturing fluid has evolved from an innovative approach into a cornerstone stimulation technique for low-permeability reservoirs worldwide [8,9,10]. Its widespread adoption is driven by three key advantages: the demonstrated ability to create complex fracture geometries, minimal formation damage, and notable cost-effectiveness. The technique fundamentally involves pumping large volumes of water at high rates and pressures to mechanically fracture the rock.

A major technical challenge arises from the high-velocity flow of fracturing fluid through extensive wellbores and tubulars, where turbulent dissipation causes significant frictional pressure loss, directly consuming pump horsepower [11,12]. This inefficiency hinders the optimal transfer of hydraulic energy to the fracture tip, potentially limiting far-field fracture propagation. Therefore, reducing flow resistance to improve downhole energy transport efficiency is paramount for optimizing treatment performance. In practice, slickwater fracturing is primarily water (and proppant) formulated with low concentrations of functional additives to ensure operational integrity and formation compatibility, including a drag reducer (DR), a biocide, scale/clay-control agents, surfactants, corrosion/oxygen-control chemicals (e.g., oxygen scavengers), and breakers when needed [13,14,15]. As the core polymeric additive in slickwater, polyacrylamide (PAM) and its derivatives perform two synergistic functions: first, as efficient drag reducers to suppress turbulence and minimize frictional losses during pumping [16,17], and second, by providing a crucial increase in fluid viscosity necessary for suspending and transporting proppants deep into the created fractures to prevent closure after pressure release. From a materials perspective, these functions are intrinsically related to the formation of transient, weak gels or gel-like polymer networks under flow and confinement conditions.

However, as the industry pushes into deeper, hotter, and more geochemically complex reservoirs, fracturing fluids face increasingly severe conditions. These environments impose stringent demands on the performance stability of PAM-based polymers. These additives must maintain their drag-reduction and proppant-carrying functions under extreme downhole conditions of high temperatures (often >120 °C) and high salinity (total dissolved solids frequently exceeding 1 × 10^4^ mg/L) [18]. Conventional linear PAM suffers from rapid performance degradation under such harsh conditions. At elevated temperatures, the polymer backbone undergoes thermal–oxidative degradation, and the amide groups hydrolyze to carboxylate groups [19,20], weakening the chain and altering its charge. In high-salinity environments, particularly in brines rich in divalent cations (e.g., Ca^2+^ and Mg^2+^), electrostatic repulsion between anionic chains is shielded [21,22], causing chain coiling, contraction, and a drastic loss of hydrodynamic volume, which decreases viscosity and drag-reduction efficacy. Consequently, enhancing the thermal stability and salt tolerance of PAM and its derivative polymers through advanced molecular design has emerged as a critical scientific challenge and a technical bottleneck for the advancement of slickwater fracturing in demanding reservoirs.

Although several recent reviews address fracturing fluids and polymer additives, they mainly provide system-level or performance-focused summaries and rarely link PAM’s molecular architecture to both drag reduction and weak-gel/viscosity enhancement under high-temperature and high-salinity conditions. This review systematically examines recent progress in the development and application of PAM-based polymers for slickwater fracturing fluids. We first outline the primary synthetic methodologies, then focus on molecular design strategies and mechanisms for improving high-temperature resistance and salt tolerance. Next, we summarize the current application status and performance of these polymers in drag reduction and gelation. Finally, we present perspectives on ongoing challenges and promising future research directions. This comprehensive review aims to provide theoretical insights and practical guidance for developing next-generation, high-performance fracturing fluid additives suitable for extreme reservoir conditions.

## 2. Synthesis Methods of Polyacrylamide

Acrylamide (AM) serves as the fundamental monomer for synthesizing polyacrylamide (PAM). It can be homopolymerized to form linear PAM or copolymerized with functional monomers to impart specific properties such as enhanced charge density, thermal stability, or salt tolerance. The choice of polymerization method critically determines the final product’s molecular architecture, physical form, and performance. The most prevalent techniques include aqueous solution polymerization, inverse emulsion polymerization, and aqueous dispersion polymerization, as summarized in Table 1 [23,24,25,26].

### 2.1. Aqueous Solution Polymerization

**Figure 2:**
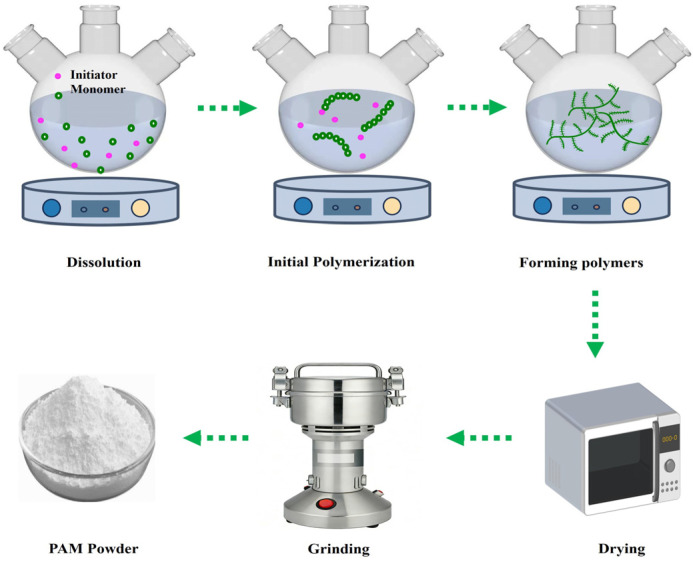
The process of synthesizing PAM via aqueous solution polymerization.

Aqueous solution polymerization is a conventional method for synthesizing PAM (Figure 2), wherein water acts as the dispersion medium and polymerization proceeds in the aqueous phase using water-soluble initiators [27,28]. Following the reaction, the product, typically obtained in a gel state, undergoes drying and pulverization to yield a solid powder. This method offers advantages such as a simple process, the ability to produce high-molecular-weight polymers, ease of transportation and storage, and environmental friendliness due to the absence of organic solvents [29,30].

However, the substantial exothermic heat released during polymerization accelerates the reaction and markedly increases solution viscosity. The high viscosity impedes heat dissipation, often causing rapid gelation into a rubber-like material. Such gels exhibit broad molecular weight distributions and poor water solubility, frequently leading to clogging of discharge ports and processing equipment [31]. Although industrial production can optimize monomer concentrations and process parameters to ensure product quality and safety, the resulting high-molecular-weight powder requires extensive on-site mixing equipment for rapid dissolution in slickwater fracturing applications [32,33]. This reduces operational efficiency and increases completion costs—key factors driving the development of emulsion-based PAM products.

### 2.2. Inverse Emulsion Polymerization

**Figure 3:**
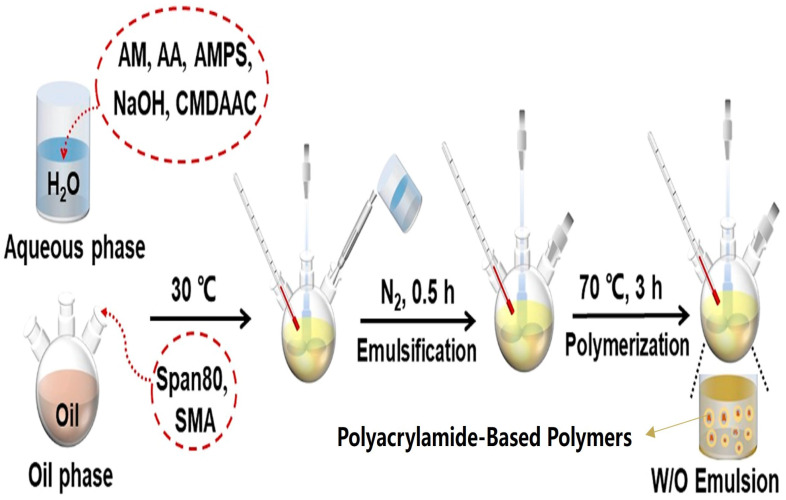
The process for copolymerizing water-in-oil-type PAM [36].

Inverse emulsion polymerization, commonly referred to as the water-in-oil (W/O) emulsion method, is an established technique for producing emulsion-type PAM [34,35]. In this process, an aqueous monomer solution is emulsified into a continuous oil phase using surfactants; polymerization then occurs within the dispersed aqueous droplets to yield a stable W/O emulsion. For example, in the synthesis of a copolymer W/O emulsion PAM (Figure 3), AM, acrylic acid (AA), 2-acrylamido-2-methylpropanesulfonic acid (AMPS), and cetylmethyldiallylammonium chloride (CMDAAC) are dissolved in an aqueous NaOH solution. Simultaneously, emulsifiers such as Span 80 and stearyl methacrylate (SMA) are dissolved in the oil phase [36]. Under vigorous stirring, the aqueous solution is added to the oil phase to form a stable emulsion, followed by polymerization within the droplets to obtain the final product.

PAM produced via this route typically possesses a moderate-to-high molecular weight with a narrow molecular weight distribution and dissolves rapidly upon inversion in water. Unlike aqueous-solution-polymerized PAM, the emulsion product requires no drying or grinding [37,38], effectively addressing the slow dissolution, large equipment footprint, and material waste associated with powdered PAM, thereby enhancing its suitability for large-scale fracturing [39,40]. However, this method demands stricter process control and involves organic solvents in the continuous oil phase, raising environmental concerns.

### 2.3. Dispersion Polymerization

**Figure 4:**
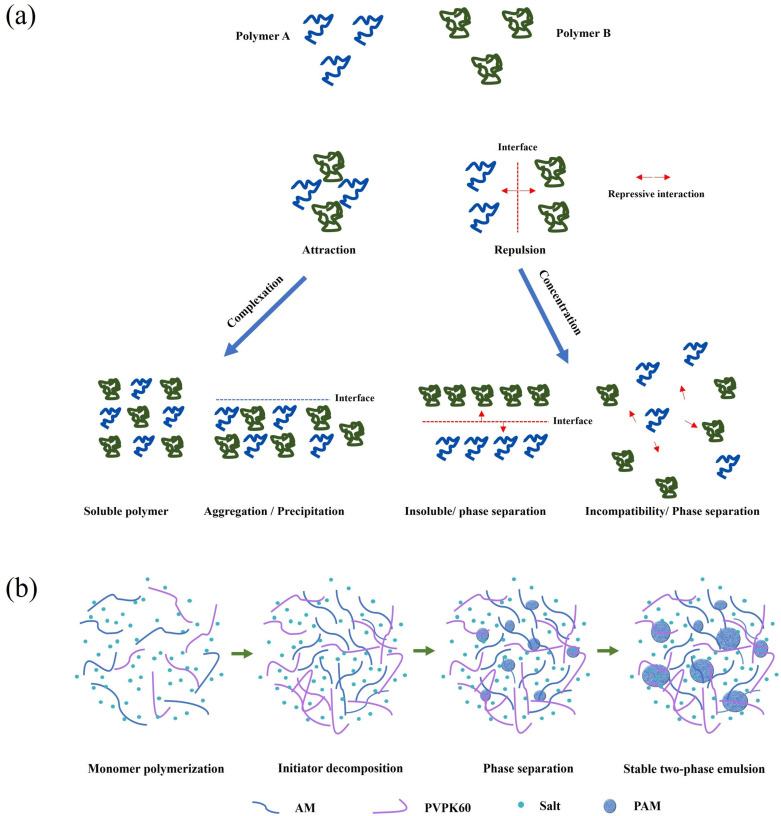
(**a**) Mechanism of water dispersion polymerization; (**b**) synthesis process of PAM via water dispersion polymerization.

Aqueous dispersion polymerization provides a solvent-free route for PAM synthesis [41,42]. Based on the phase-separation mechanism, the systems are primarily classified into two categories: (1) the PAM-based polymer-polymer–water (aqueous two-phase) system, in which mixing two water-soluble polymers (e.g., polyethylene glycol (PEG) or polyvinylpyrrolidone (PVP)) leads to phase separation due to a positive Gibbs free energy change (Figure 4a), and (2) the PAM–salt-water system, which relies on the salting-out effect induced by inorganic salts such as sulfates or phosphates [43].

The former system entails higher synthesis costs, while the latter often results in excessively rapid phase separation, hindering the formation of high-molecular-weight products. A hybrid approach can combine the advantages of both. As illustrated in Figure 4b, the process comprises four stages: monomer dissolution, initial polymerization, polymer precipitation, and system stabilization [44]. Specifically, AM, inorganic salts (e.g., NaCl and (NH_4_)_2_SO_4_), and a stabilizer (e.g., polyvinylpyrrolidone) are dissolved to form a homogeneous solution. After temperature adjustment, an initiator such as (NH_4_)_2_S_2_O_8_/NaHSO_3_ is added to initiate polymerization. As the molecular weight increases, the nascent polymer precipitates as particles via the salting-out process, forming a dispersed phase. Polymerization then continues on the particle surface, yielding a stable water-in-water (W/W) emulsion.

W/W emulsion PAM offers rapid dissolution, low system viscosity, no oil-phase contamination, and a relatively low cost [45,46,47]. However, its storage stability is generally limited, and the achievable molecular weight is comparatively low, making it more suitable for environmentally conscious fracturing operations.

## 3. Methods for Enhancing Thermal Stability

When dissolved in water, PAM significantly increases the viscosity of the aqueous phase and reduces flow resistance, characteristics that underpin its dual functionality in fracturing fluids [48,49,50]. However, in deep oil and gas wells with wellbore lengths exceeding 3500 m, the molecular backbone of conventional PAM is susceptible to degradation at elevated temperatures (>80 °C), leading to a marked decline in performance [51,52,53]. Consequently, improving the thermal stability of PAM has become a central research focus. Current strategies to enhance its temperature tolerance primarily include synthesizing ultra-high-molecular-weight PAM (UHMW-PAM), copolymerization with thermally stable monomers, and the incorporation of nanoparticles into the polymer matrix.

### 3.1. Synthesis of Ultra-High-Molecular-Weight Polyacrylamide

**Figure 5:**
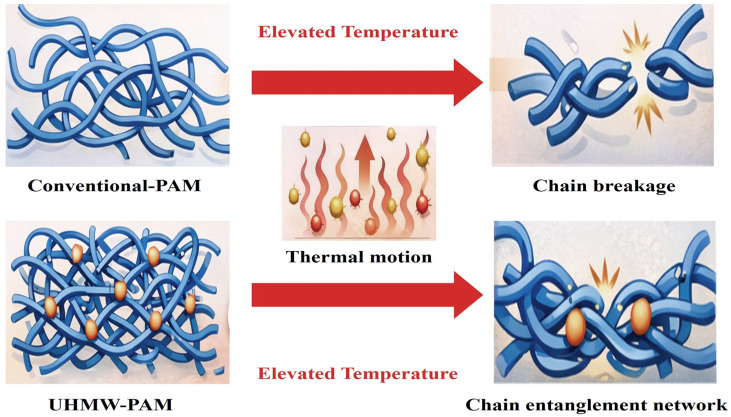
The temperature resistance mechanism of ultra-high-molecular-weight polyacrylamide.

The enhanced thermal resistance of UHMW-PAM originates from its substantially extended polymer chain length [54,55,56]. In the ultra-high-molecular-weight regime, individual macromolecules exhibit high flexibility and extensibility, forming dense chain entanglements in a solution. These long chains construct a physically cross-linked, gel-like network (often referred to as a weak or physical gel) via entanglement, which effectively preserves the structural integrity of the gel-like network under thermal stress, as shown in Figure 5. At elevated temperatures, intensified molecular motion increases chain-segment mobility and local tension, raising the risk of chain scission [57,58]. However, strong entanglement and frictional interactions between long chains in UHMW-PAM restrict segmental movement, thereby stabilizing the gel-like network structure and enabling the polymer to retain its structural integrity under high-temperature conditions [59,60]. Consequently, UHMW-PAM exhibits superior thermal stability, shear resistance, and viscosity retention compared to conventional PAM.

Currently, UHMW-PAM is primarily synthesized via aqueous solution polymerization and inverse emulsion polymerization. Aqueous solution polymerization, characterized by simple operation, can yield polymers with molecular weights exceeding 1 × 10^7^ by precisely controlling reaction parameters [61,62,63]. For instance, Wang et al. [64] synthesized powdered UHMW-PAM with a viscosity-average molecular weight (*M*η) of 4.25 × 10^7^ via aqueous copolymerization. This polymer forms a stable three-dimensional entangled network, exhibiting characteristic gel-like rheological behavior, and demonstrating excellent thermal and shear resistance. Subsequent refinements in the polymerization process have yielded UHMW-PAM with an *M*η of 4.97 × 10^7^ under mild aerobic conditions, highlighting the potential of this method to push the molecular-weight limit [65].

In contrast, inverse emulsion polymerization typically produces emulsion-type UHMW-PAM with somewhat lower molecular weights but offers advantages such as rapid dissolution and operational convenience. For example, Yang et al. [66] prepared an emulsion-type UHMW-PAM with an *M*η of 1.5 × 10^7^ via inverse emulsion polymerization. Although the molecular weight is lower than that from aqueous polymerization, the fast solubility and ease of use make it valuable for field applications. Key limitations for further molecular weight increases in this system include diffusion constraints within aqueous droplets and chain-transfer reactions at the oil–water interface. Therefore, minimizing interfacial effects and enhancing intra-droplet chain propagation are important research directions for improving emulsion-type UHMW-PAM.

### 3.2. Copolymerization with Thermally Resistant Monomers

Copolymerizing acrylamide (AM) with thermally stable monomers to introduce bulky or rigid side groups into the PAM backbone is an effective strategy to enhance thermal stability. The mechanism involves these side groups increasing resistance to segmental motion and inhibiting intramolecular rotation, thereby stiffening the polymer chains and improving their resistance to high-temperature degradation [67,68,69].

**Figure 6:**
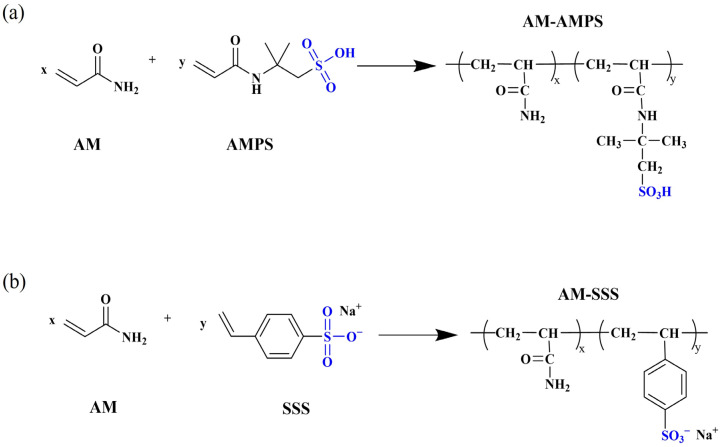
(**a**) AM-AMPS structure formula; (**b**) AM-SSS structure formula.

Common thermally stable comonomers include sulfonic acid-group-containing monomers (e.g., AMPS and sodium p-styrenesulfonate (SSS)) and cyclic-structure-containing monomers (Table 2). Among these, sulfonic acid-based monomers have been most extensively studied. However, the thermal stability of AM-AMPS copolymers (Figure 6a) is typically limited to temperatures below 100 °C, primarily due to the hydrolysis of the amide group in AMPS at elevated temperatures [70], which leads to sulfonate loss and chain destabilization. In contrast, AM-SSS copolymers (Figure 6b) feature a benzene ring in the side chain; the benzenesulfonate structure confers higher thermal stability, although the hydrophobic benzene ring may reduce copolymer solubility. For instance, the AM-SSS copolymer synthesized by Borai et al. [71] withstood temperatures up to 120 °C. This is attributed to a robust hydrogen-bonding network formed between sulfonate groups and the carbonyl/amino groups of the acrylamide units, which gives rise to a transient, gel-like associative structure, enhancing intermolecular forces and resisting thermal chain scission.

In summary, introducing rigid or associative side groups is a key strategy for improving PAM’s thermal stability, as these modifications reinforce gel-like or weakly associated polymer networks that remain stable under elevated temperatures. Future research should focus on developing novel monomers that combine high thermal stability with good water solubility or explore synergistic effects in multimonomer copolymerization.

### 3.3. Incorporation of Nanoparticles

Incorporating nanoparticles into PAM forms nanocomposite gel-like systems, where PAM is the continuous phase and nanoparticles are uniformly dispersed [76,77,78]. The nanophase induces strong interactions with the polymer matrix, acting as physical cross-linking points that reinforce the gel-like polymer network, thereby enhancing the thermal resistance of the composite [79,80,81]. For example, Liu et al. [82] incorporated highly surface-active nano-silica into PAM. The resulting composite maintained good thermal stability at 90 °C, primarily due to hydrogen bonding between PAM amide groups and silica silanol groups, which extends the effective chain length and reinforces the physically cross-linked network and the gel-like network.

**Figure 7:**
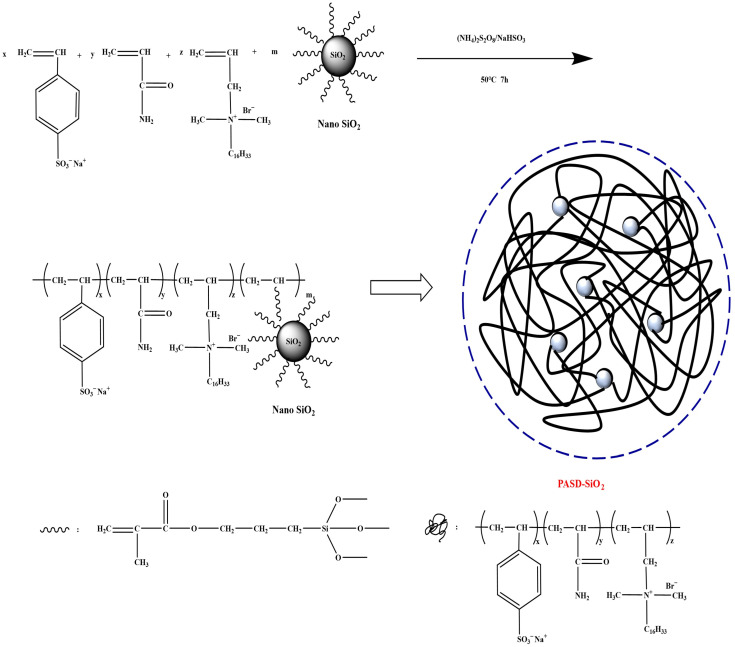
Synthesis route for the nanocomposite polymer PASD-SiO_2_ [83].

Conventional nanoparticles tend to agglomerate, hindering uniform dispersion and sufficient interaction with the polymer. Surface modification is often required to improve dispersibility and interfacial compatibility. For instance, Xing et al. [83] modified silica nanoparticles with γ-methacryloxypropyltrimethoxysilane (KH-570) and grafted them onto PAM chains via aqueous solution polymerization (Figure 7). The resulting nanocomposite exhibited enhanced thermal resistance, shear tolerance, and salt tolerance compared to neat PAM, mainly due to the uniform dispersion of the modified nanoparticles and their crosslinker-like effect, giving rise to a pseudo-cross-linked nanocomposite gel structure.

In summary, nanocomposite formation is an effective approach to improving the thermal stability of PAM, as nanoparticles function as physical or pseudo-cross-linking nodes that stabilize gel-like polymer networks under thermal stress. Future research should investigate the long-term performance mechanisms of PAM nanocomposites under high-temperature reservoir conditions. However, performance enhancement does not always yield purely positive outcomes. The incorporation of nanoparticles may compromise the intrinsic activity of native PAM-based additives (e.g., drag reduction). Although strong polymer–polymer or polymer–nanoparticle interactions significantly improve thermal and salt tolerance, excessive cross-linking density, rigid composite structures, or overly strong associative interactions can restrict chain flexibility and inhibit shear-induced molecular elongation—both critical for turbulent drag reduction. Thus, a trade-off exists between improving environmental tolerance and preserving the intrinsic activity of PAM-based drag reducers, underscoring the need for rational molecular design to balance stability, rheological behavior, and flow-induced functionality.

## 4. Methods for Enhancing Salt Resistance

**Figure 8:**
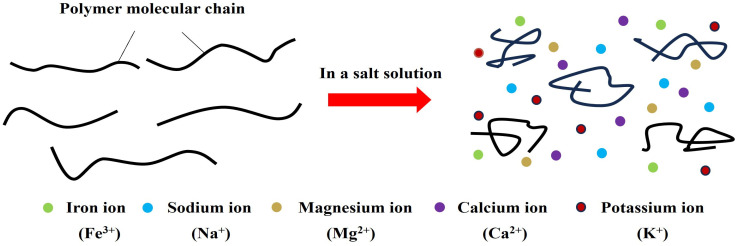
The influence of salt water on the molecular chain of conventional drag reducers.

Conventional slickwater fracturing requires substantial freshwater, severely limiting its use in water-scarce regions [84,85,86]. Consequently, formulating fluids with alternative water sources (e.g., flowback water and seawater) has become a technical priority. However, in high-salinity brines (≥1 × 10^4^ mg/L), PAM chains undergo coiling (Figure 8), leading to a drastic loss of viscosity and drag reduction, which can cause operational failure [87,88,89]. Improving PAM’s salt tolerance is therefore critical. The principal strategy involves grafting charged side groups (anionic or cationic) onto its backbone (Table 3). These groups are less sensitive to salt ions and maintain an extended chain conformation in brine via electrostatic repulsion, effectively inhibiting salt-induced chain collapse.

### 4.1. Grafting Anionic Side Groups

**Figure 9:**
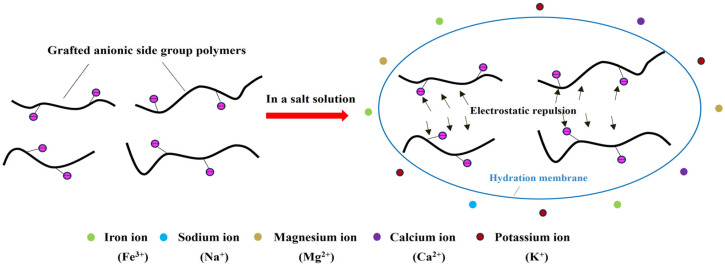
The salt tolerance mechanism of grafted anionic side group polymers.

Grafting anionic groups (e.g., carboxylate, sulfonate) onto PAM is a common method to enhance salt tolerance [96,97]. The mechanism involves electrostatic repulsion and hydration-layer stabilization (Figure 9). Ionized anionic groups create repulsive forces between chains, preventing coiling and maintaining an extended conformation in saline media [98,99,100]. Concurrently, anions in a solution form a hydration shell around the charged polymer chains, shielding them from cation-induced charge neutralization and further suppressing collapse [101,102].

Common anionic monomers include AMPS, acrylic acid (AA), and sodium vinyl sulfonate. AMPS, for instance, improves both thermal stability and salt tolerance. Chen et al. [85] synthesized an AM/AMPS copolymer emulsion that met fracturing performance requirements at 0.2 wt% in high-salinity brine (2 × 10^4^ mg/L, containing Fe^3+^). To achieve higher salt resistance, copolymers incorporating both AA and AMPS have been developed. The combined carboxylate and sulfonate groups strengthen interchain electrostatic repulsion, maintaining an extended chain conformation at salinities up to 3 × 10^4^ mg/L [103]. However, excessive anionic groups can increase steric hindrance during polymerization, inhibit chain growth, reduce molecular weight, and raise costs.

In summary, grafting anionic side groups effectively enhances salt tolerance, but their structure and concentration must balance performance gains with synthetic feasibility. Future work should optimize monomer ratios and polymerization processes to synergistically improve salt tolerance, molecular weight, and cost-effectiveness.

### 4.2. Grafting Cationic Side Groups

Incorporating cationic monomers (e.g., quaternary ammonium salts) imparts a positive charge to PAM chains. In saline solutions, these chains adsorb onto negatively charged suspended particles, neutralizing their charge [104,105,106]. A single PAM chain can then bridge multiple neutralized particles, forming compact, stable flocs. This process inhibits chain coiling and helps maintain an extended conformation.

Common cationic monomers include dimethylaminoethyl methacrylate (DMAEMA), methacryloyloxyethyltrimethylammonium chloride (DMC), and acryloyloxyethyltrimethylammonium chloride (DAC). DMC is widely used due to its high reactivity, permanent positive charge across a wide pH range, good solubility, and salt tolerance. Ma et al. [92] introduced quaternary ammonium groups into PAM via inverse emulsion polymerization, producing a water-in-oil (W/O) emulsion. The cationic groups enhanced non-covalent interchain interactions, allowing the emulsion to retain good viscosity-enhancing properties at 3 × 10^4^ mg/L salinity. Field applications showed improved flowback water utilization and reduced formation damage.

Thus, introducing cationic groups is a viable strategy for improving salt tolerance. Future research should focus on optimizing cationic monomer structure and elucidating structure–performance relationships in complex saline environments.

### 4.3. Grafting Amphoteric Side Groups

Simultaneously introducing anionic and cationic side groups yields amphoteric PAM copolymers. The coexistence of opposite charges on the same chain creates intramolecular or intermolecular electrostatic attractions, forming dynamic cross-links or compact conformations [107,108]. These internal interactions counteract the shielding effect of external salt ions, helping maintain an extended chain conformation and thus preserving viscosity-enhancing function [109,110,111]. For example, Dai et al. [95] synthesized an amphoteric PAM by grafting both sulfonate and quaternary ammonium groups, which retained viscosity-building capacity in ultra-high-salinity brine (1.6 × 10^5^ mg/L) and showed 39% viscosity retention at 120 °C.

The synthesis of amphoteric PAM is challenging, as the positive-to-negative charge ratio must be precisely controlled to balance solubility, conformation, and performance, leading to complex processes and higher costs. Therefore, amphoteric PAMs are still under development. Future work should explore the structure–performance relationships governing charge distribution and chain architecture under reservoir conditions to facilitate industrial application.

Polyacrylamide (PAM) and its derivatives are widely used in slickwater fracturing fluids, primarily serving as drag reducers and viscosity enhancers [112,113]. As drag reducers, their core function is to suppress turbulent frictional resistance during high-velocity pumping, effectively lowering wellhead pressure and improving fluid-transport efficiency. As viscosity enhancers, they increase the system’s viscosity to suspend and carry proppants (e.g., ceramic or sand) deep into fractures, thereby propping open the fractures, enhancing network conductivity, and ultimately improving hydrocarbon recovery.

## 5. Drag Reduction in Slickwater Fracturing Fluid

### 5.1. Mechanisms of Drag Reduction

**Figure 10:**
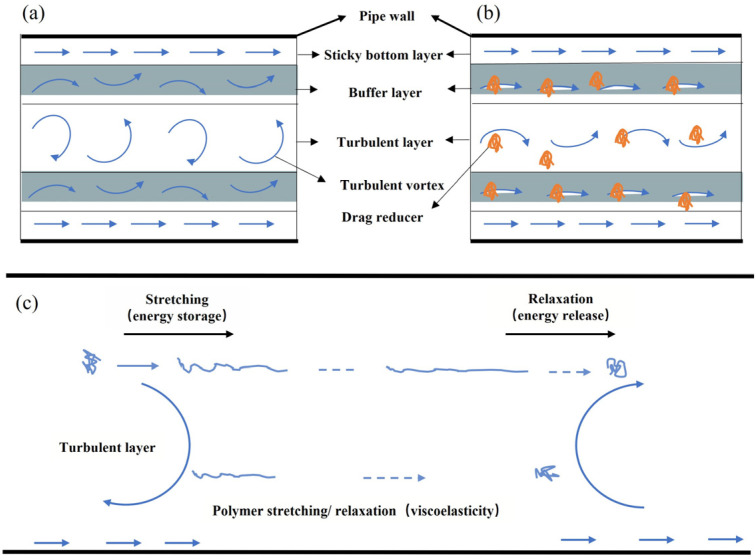
Turbulence suppression theory and viscoelastic theory mechanisms for reducing drag. (**a**) No drag reducer [125]. (**b**) There is a drag reducer [125]. (**c**) Schematic representation of the elastic drag-reduction mechanism.

PAM significantly reduces flow resistance in pipelines [114,115]. Two principal theories explain this effect:

(1) Turbulence suppression theory: Fluid flow in a pipe is divided into three regions (Figure 10a): the viscous sublayer (laminar, low energy dissipation), the buffer layer (small-scale turbulence), and the turbulent core (highly disordered, large energy dissipation) [116,117]. Extended PAM chains dissolved in the fluid suppress the transverse momentum exchange perpendicular to the main flow direction (Figure 10b), thereby reducing turbulence intensity and frictional resistance [118,119,120]. This theory, based on momentum transfer, is widely accepted.
(2) Viscoelasticity theory: This mechanism emphasizes the interaction between viscoelastic polymer chains and near-wall turbulent structures [121,122]. In regions with a high strain rate, polymer coils are stretched toward extended conformations and temporarily store part of the turbulent kinetic energy as elastic/entropic energy (Figure 10c). As the stretched chains are convected into regions of lower strain, they relax back toward coiled conformations and release the stored energy to the flow, which weakens near-wall vortical motions and reduces turbulent energy dissipation [123,124].

As schematically illustrated in Figure 10, the turbulence suppression mechanism primarily operates by reducing transverse momentum exchange in the buffer and turbulent layers, whereas the viscoelastic mechanism involves elastic energy storage and release by stretched polymer chains interacting with near-wall vortical structures. Together, these two mechanisms explain the drag-reduction behavior of PAM-based slickwater systems under turbulent flow conditions.

### 5.2. Evolution of Drag Reducers

**Figure 11:**
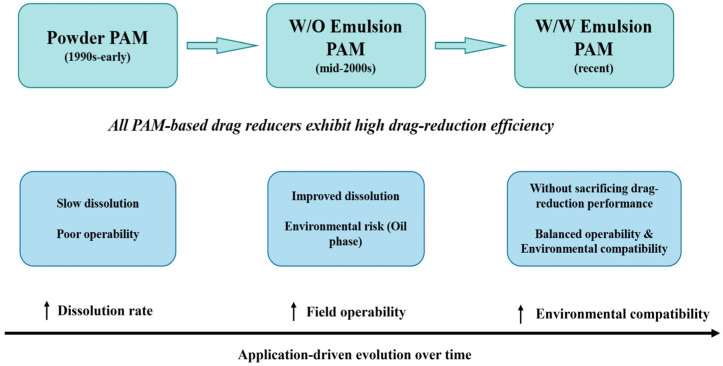
Application-driven evolution of PAM-based drag reducers for slickwater fracturing.

The evolution of PAM-based drag reducers has been driven not only by the pursuit of high drag-reduction efficiency but also by the need for rapid dissolution, field operability, and environmental compatibility, as schematically summarized in Figure 11.

Efficient drag reduction requires PAM to dissolve rapidly and maintain an extended, elastic chain conformation under combined high-temperature, high-salinity, and high-shear conditions. High-molecular-weight (HMW) PAM with linear or weakly branched structures provides a large hydrodynamic radius, effectively suppressing near-wall eddies and reducing friction [126,127,128]. However, HMW PAM often dissolves slowly and forms insoluble “fish-eyes,” limiting its use in large-scale, high-rate fracturing.

Unlike dense powders from aqueous solution polymerization, drag reducers made by inverse emulsion or aqueous dispersion polymerization contain active components such as micro-/nano-scale “pre-swollen” particles. This morphology drastically shortens dissolution time, enabling real-time mixing at the wellsite. Consequently, product forms have evolved from powders to emulsions. For example, Zhi et al. [129] developed an imidazole-functionalized W/O drag reducer via inverse emulsion polymerization. At pH 2.75 and a 0.06% dosage, it achieved 73% drag reduction within 150 s and showed good storage stability, and it has been used in unconventional reservoirs. However, its oil phase poses risks of formation damage and environmental contamination.

Therefore, environmentally friendly water-in-water (W/W) emulsions have become a key R&D focus. For instance, Zhao et al. [44] prepared a W/W drag reducer via aqueous dispersion polymerization (*M*η = 1.9–2.4 × 10^6^; dissolution time = 24 s). At a 0.1% concentration and a 30 L/min flow rate, it achieved 73.08% drag reduction with <10% formation damage, balancing high performance with environmental compatibility.

Furthermore, introducing suitable ionic or hydrophilic groups can tune chain rigidity, charge density, and solvation. This helps suppress chain entanglement and promotes chain extension under shear, maximizing turbulence suppression. To sustain performance under high-temperature, high-salinity conditions, it is essential to incorporate temperature-/salt-tolerant monomers or construct dynamic physical cross-linking networks.

## 6. Gelation in Slickwater Fracturing Fluid

Early PAM applications focused on reducing wellbore friction, but conventional low-viscosity solutions lacked sufficient proppant-carrying capacity for effective fracture creation and conductivity maintenance [130,131]. As operations extend into deeper reservoirs with higher closure stress, fluids must deliver high proppant concentrations to form and sustain conductive fracture networks [132,133]. The key requirement for thickeners is to rapidly form a robust, temperature-/salt-/shear-resistant viscoelastic network at ultra-low concentrations. PAM design has thus advanced from simple linear chains to strategies based on hydrophobic association and chemical cross-linking [134,135,136].

### 6.1. Chemical Cross-Linking with Crosslinkers

**Figure 12:**
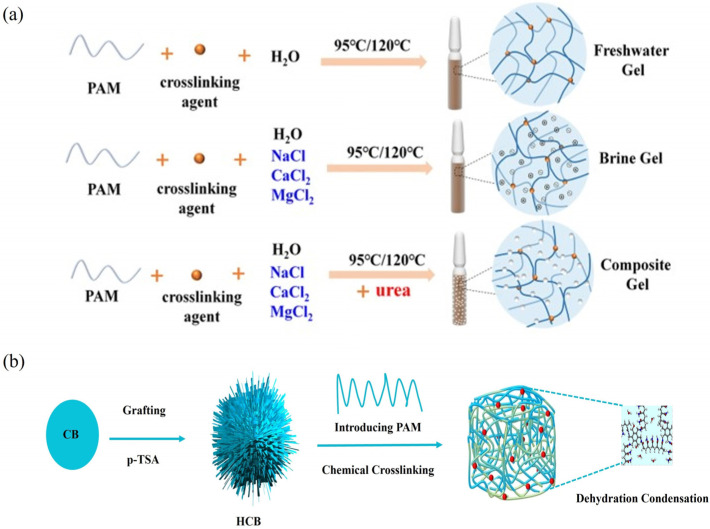
(**a**) PAM cross-linking mechanism [138]; (**b**) HCB/PAM nanocomposite gel.

In chemical cross-linking (Figure 12a), cross-linkers (e.g., multivalent metal ions or organic agents) react with active groups (e.g., –COOH and –CONH_2_) on PAM chains, forming covalent/ionic bonds that connect them into a stable 3D gel network [137,138]. This network severely restricts chain motion, increasing viscosity by orders of magnitude and imparting excellent viscoelasticity. The strong, elastic gel can suspend high proppant loads and withstand formation pressures, which is crucial for long-term fracture conductivity. Key considerations are cross-linker selection and density control. Two common types include

(1) Multivalent metal ions: Ge et al. [139] combined a metal cross-linker with hydrophobically associating PAM. The system acts as a drag reducer at low concentrations but transforms into a heat-stable gel (80 mPa·s after 1 h at 120 °C, 170 s^−1^) via coordination cross-linking at higher concentrations, enabling intelligent “drag-reduction-to-viscosity-enhancement” switching.
(2) Surface-functionalized nanoparticles: Bai et al. [140] grafted hyperbranched polyester onto nano-carbon black and covalently cross-linked it with PAM chains, producing an HCB/PAM nanocomposite gel (Figure 12b). The nano-carbon black acts as a rigid nano-cross-linker, significantly boosting thermal stability (withstands 200 °C) and mechanical strength, thereby improving proppant transport and reducing formation damage.

### 6.2. Physical Cross-Linking via Hydrophobic Association

**Figure 13:**
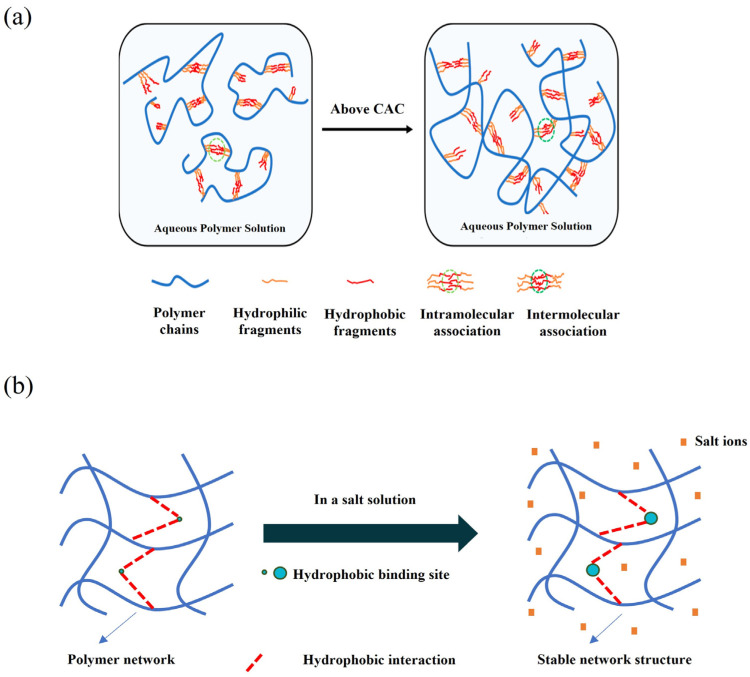
(**a**) The thickening mechanism for a hydrophobic associating polymer [143]. (**b**) Salt ion-induced enhancement of hydrophobic interactions in polymers.

Hydrophobic association (Figure 13a) involves introducing a small fraction of hydrophobic monomers (e.g., long-chain alkyl acrylates) into the PAM backbone to promote dynamic intramolecular/intermolecular aggregation of hydrophobic groups in water, thereby forming reversible physical cross-links [132,133]. This creates a transient 3D network that significantly increases hydrodynamic volume and structural viscosity, even at low polymer concentrations. The network also provides beneficial shear-thinning and recovery properties, ensuring efficient proppant transport and placement.

Hydrophobic association is achieved by grafting controlled amounts of specific hydrophobic groups (e.g., C_12_ alkyl, phenyl, and APO-10) onto the PAM backbone. In water, these groups dynamically associate to form reversible physical cross-links, building a stable 3D network at low concentrations. For example, Dai et al. [141] synthesized an APO-10 copolymer whose hydrophobic association is enhanced by salt ions, yielding a viscosity of 35 mPa·s at only 0.04 wt% (Figure 13b). To improve association kinetics, monomers with dual hydrophobic tails and hydrophilic spacers (e.g., PEO chains) have been designed. Zhang et al. [142] developed such a dual-tailed polymer that forms a dense network at 0.03 wt%, exhibiting excellent salt and shear resistance.

## 7. Conclusions

This review provides an overview of the development and application of polyacrylamide (PAM)-based polymers in slickwater fracturing fluids, focusing on synthesis routes, molecular design strategies for high-temperature (>120 °C) and high-salinity (>1 × 10^4^ mg/L) reservoirs, and the dual functions of drag reduction and weak-gel/viscosity enhancement. The main conclusions are as follows:

(1) The principal synthesis routes for PAM-based slickwater additives include aqueous solution polymerization, inverse emulsion polymerization, and aqueous dispersion polymerization, which determine product form and field operability.
(2) Thermal stability can be enhanced by synthesizing ultra-high-molecular-weight PAM, copolymerizing with thermally resistant monomers, and incorporating nanoparticles to reinforce gel-like polymer networks.
(3) Salt tolerance can be improved by grafting anionic, cationic, or zwitterionic side chains onto the polymer backbone to mitigate salt-induced chain collapse and preserve chain conformation in brines.
(4) PAM-based additives provide dual functionality in slickwater: drag reduction to lower turbulent friction during high-rate pumping and viscosity/weak-gel enhancement to improve proppant transport and fracture conductivity.
(5) Water-in-water (W/W) PAM emulsions prepared via aqueous dispersion polymerization show clear advantages in rapid dissolution and environmental compatibility, but storage stability and achievable molecular weight remain key limitations.
(6) Strengthening associative or (pseudo-)cross-linked structures can improve tolerance to harsh conditions, yet excessive interactions may restrict chain flexibility and shear-induced elongation, potentially compromising drag-reduction efficiency; thus, stability and flow functionality must be balanced in design.

## 8. Discussion

For practical deployment in deep and complex reservoirs, performance improvements must be weighed against cost, scalability, environmental impact, and long-term reliability. Compared with oil-phase emulsions and highly complex hybrid systems, W/W emulsions appear more field-realistic because they better support rapid on-site mixing and align with environmental constraints. However, the long-term stability of PAM under combined high-temperature, high-salinity, and pumping-induced shear conditions is not yet well established, making it difficult to reliably predict sustained drag reduction and weak-gel/viscosity performance in the field.

## Figures and Tables

**Table 1:** Characteristics of PAM synthesized via three different polymerization methods.

Polymerization Method	Reaction Medium	Initiation System	Product Morphology	Polymer Molecular Weight	Solubility	Stability	Reference
Aqueous Solution Polymerization	Water	Water-soluble	Gel	High, Broad Distribution	Slow	Excellent	[23,24]
Inverse Emulsion Polymerization	Oil–water	Oil-soluble	Oil-in-water emulsion	Medium, Narrow Distribution	Fast	Good	[25]
Water Dispersion Polymerization	Alcohol–water/salt–water	Water-soluble	Water-in-water emulsion	Low, Uniform, Controllable	Faster	Poor	[26]

**Table 2:** Different types of temperature-resistant monomers.

Type	Name	Structural Formula	Key Structural Feature	Mechanism of Action	Reference
Sulfonate monomer	2-Acrylanmido-2-methylpropanesulfonic acid (AMPS)	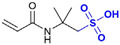	Bulky sulfonated tert-carbon	Steric hindrance and strong hydration restrict chain motion and thermal degradation.	[72]
	Sodium p-Styrene Sulfonate (SSS)	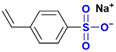	Aromatic sulfonated ring	Rigid aromatic rings increase chain stiffness and thermal stability.	[37]
	2-Methacrylamido-2-methylpropanesulfonic acid (MAMPS)	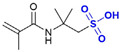	α-Methyl + sulfonate group	α-Methyl substitution and sulfonated bulk synergistically reduce chain flexibility.	[73]
Monomers containing cyclic structures	N-vinylpyrrolidone (NVP)	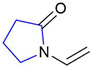	Five-membered lactam ring	Rigid lactam rings restrict chain motion and form stabilizing hydrogen bonds.	[74]
	N-Vinylcaprolactam (NVCL)	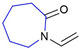	Seven-membered lactam ring	Larger cyclic lactam rings enhance chain rigidity and intermolecular interactions.	[75]

**Table 3:** Comparison of ionic side groups for improving salt tolerance of polyacrylamide.

Ionic Side Group Type	Representative Groups/Monomers	Salt-Tolerance Mechanism	Advantages	Disadvantages	Reference
Anionic side groups	Carboxylate (–COO^−^ and AA); sulfonate (–SO_3_^−^, AMPS, and SVS)	Electrostatic repulsion between negatively charged chains suppresses chain coiling; hydrated shells shield charges from cation neutralization.	Mature and well-understood strategy; effective at moderate–high salinity	Excess anionic content increases steric hindrance; reduced polymerization degree and molecular weight	[90,91]
Cationic side groups	Quaternary ammonium (DMC, DAC); amino groups (DMAEMA)	Positive charges neutralize negatively charged particles; polymer bridging forms stable flocs and suppresses excessive chain contraction.	Good salt tolerance in complex brines; Enhances flowback-water reuse	Strong interactions with suspended solids and minerals; increased system complexity	[92,93]
Amphoteric side groups	Sulfonate + quaternary ammonium (e.g., PASD)	Intramolecular/intermolecular electrostatic attraction forms dynamic cross-linking structures, counteracting external ionic shielding.	Superior salt tolerance; best adaptability to complex brines	Complex synthesis and charge-ratio control; high production cost	[94,95]

## Data Availability

Data are contained within the article.

## Abbreviations

PAM	Polyacrylamide
W/O	Water-in-oil
W/W	Water-in-water
UHMW-PAM	Ultra-high-molecular-weight polyacrylamide
*M*η	Viscosity-average molecular weight
AMPS	2-acrylamido-2-methylpropane sulfonic acid
SSS	Sodium p-styrene sulfonate
PASD	Ampholytic polyacrylamide
KH570	3-Methacryloxypropyltrimethoxy-silane
SMA	Stearic acid methyl acrylate
DMAEMA	Dimethylaminoethyl methacrylate
DMC	Methacryloyloxyethyltrimethylammonium chloride
APO-10	Octylphenol ethoxylate (10 EO) acrylate
